# Selective detection of volatile organic compounds in microfluidic gas detectors based on “like dissolves like”

**DOI:** 10.1038/s41598-018-36615-6

**Published:** 2019-01-17

**Authors:** Mohammad Paknahad, Carmen Mcintosh, Mina Hoorfar

**Affiliations:** University of British Columbia, School of Engineering, Kelowna, Canada

## Abstract

This paper studies the effect of channel coating hydrophobicity and analyte polarity on the gas detection capability of a microfluidic-based gas detector. Two detectors with two different channel surface coating combinations (resulting in different levels of hydrophobicity) are fabricated and tested against seven analytes with different polarities (methanol, ethanol, 1-propanol, 2-pentanol, acetone, pentane, and hexane). A feature extraction method is utilized to compare the discrimination capability of each of the fabricated detector. The analysis of the combined feature space presented for both detectors reveals that the Euclidean distance, which is an indicator of the device discrimination capability between different gases, between the feature vectors of the two sensors are greater for non-polar gases compared to those obtained for the polar ones. This shows that the analyte discrimination in microfluidic gas detectors is not a purely diffusion-based process, and there are analyte/channel surface interaction parameters involved in enhancing/impeding sensor selectivity. To understand these effects, the surface free energy of each fabricated channel was determined. It is shown that the difference between the solid-liquid surface tension values estimated for the two channel surfaces is higher for the non-polar analytes as compared to the polar analytes. This effect along with the low diffusion coefficients of non-polar analyte magnifies adsorption of the analytes in the diffusion-physisorption process, resulting in a greater difference in Euclidean distances between the features obtained from the two detectors responses against non-polar analytes as compared to the polar ones. This shows that the choice of the detector**’**s channel coating material plays a key role in the selectivity of the device between different gases. As a result, non-polar channel coating surfaces are suggested for better classification of the non-polar gases, and it is shown in the cases of polar gases changing the coating surface has less effect.

## Introduction

The olfaction system^1,2^ or sense of smell is one of the most important human (and animals) abilities. Our nose affects the quality of our life significantly: detection of toxic gases in the environment, fire awareness, identifying spoiled food, good feelings initiated by pleasant smells, and memories triggered by different scents are among numerous examples. There has always been interests in developing devices that can mimic animals and human olfaction systems (similar to other sensory devices like machine vision^3^, machine hearing^4^, machine speaking^5^, and electronic tongue^6^). Mass spectrometry (MS) and gas chromatography (GC) are examples of highly accurate systems developed as machine olfaction systems^7,8^. Despite their accuracy, the size and cost of these systems limit their applicability in detection of volatile organic compounds (VOCs)^8^ in numerous applications requiring portable and easy-to-use devices (such as frequent safety and environmental monitoring^9^, food and beverage quality assessment^10^, and medical monitoring and diagnostic devices^11–13^). There have been efforts towards miniaturization of these devices (MS and GC)^14,15^. Even the handheld devices developed using GC detectors (photoionization, catalytic bead, and flame ionization) suffer from inability to detect small molecules^16^, susceptibility to poisoning^17^, and low sensitivity^18^. To alleviate the portability and other issues associated with these systems, electronic noses or e-noses (similar to the human olfactory system) have been developed^19–23^. E-noses work based on an array of sensors coupled with a pattern recognition systems. However, as a result of using a large number of sensors, these devices are expensive in terms of both the costs of the sensors and maintenance and calibration that is required due to the drifts of the components of the sensor array^24–26^. A single-sensor gas detector^27^ is an alternative as long as it provides high level of selectivity obtained from e-noses for a wide range of target gases. Recently, it has been shown that molecular diffusion of gases along a microchannel (with a high surface area to volume ratio) integrated with a single gas sensor significantly increases selectivity of a single-sensor gas detector due to diffusion-physisorption of gas molecules^28–35^. Moreover, it has been shown that optimizing the microchannel geometry and surface treatment (i.e., a combination of gold, chromium, and Parylene C) can even further enhance selectivity and sensor (channel) recovery after exposure^28^. Despite these enhancements, the interaction between the target gases and surface of the microchannel has not yet been fully understood and determined in a quantitative manner. More specifically, the effect of the channel coating (especially its correlation with the polarity of the target gas) on differentiation of different components of binary or complex gas mixtures is still unknown. Understanding these interactions is crucial for determination of an optimum coating^36^ (directly affecting selectivity of the sensor) for different target gases.

In this paper, the effect of channel hydrophobicity on the selectivity of the microfluidic-based gas detector for different VOCs are studied. Two different channel coating combinations with two different levels of hydrophobicity are compared. These coating combinations are referred to as (i) Detector O which includes gold, chromium, Parylene C, and (ii) Detector X containing gold, chromium, Parylene C, Cytonix. Cytonix (purchased from Cytonix, LLC, Product: PFCM 1104 V) is a fluoropolymer and transparent film with great hydrophobic and oleophobic properties. A variety of target gases from different families of VOCs (including alcohols (methanol, ethanol, 1-propnaol, 2-pentanol), ketones (acetone), and alkanes (pentane, hexane)) are chosen for this study. The goal is to show how the classical “similia similibus solvuntur” or “like dissolves like” principle is applied here. In essence, the differentiation of compounds along the microchannel of a microfluidic-based gas detector^28^ is based on different strengths between the interactions of the gas compounds with the surfaces the channel walls (“like dissolves like” rule). The stronger this interaction the longer the time for the analyte to migrate through the channel (by the mode of diffusion) and reach the sensor. To study the effect of this interaction, three methods are used: first, the responses of the two detectors and their selectivity has been compared (using the normalized and feature space responses of these two detectors to a range of different target gases). It is shown that these two detectors act quite differently in response to non-polar targets in comparison to polar gases. Then, a second study has been conducted on the effects of channel coating and analyte polarity on the detectors responses. Finally, the channel surface free energy of these two fabricated detectors has been determined to quantify the analyte/channel interaction and verify what it has been observed from the previous two methods. The result of this paper can be used for proper selection of channel coating based on the polarity of the target analytes.

## Results and Discussion

In this section, the transient responses recorded using the two fabricated detectors (X and O) are presented. A feature extraction method is then applied to the transient responses to compare selectivity of the two detectors using the Euclidean distances of features in the feature space. Following the characterization of channel coating and its polarity for each of the detectors, the interaction between the analyte and the surface of the microchannel is quantified based on the surface free energy of the detector channel surfaces.

### Sensor response and selectivity

**Figure 1:**
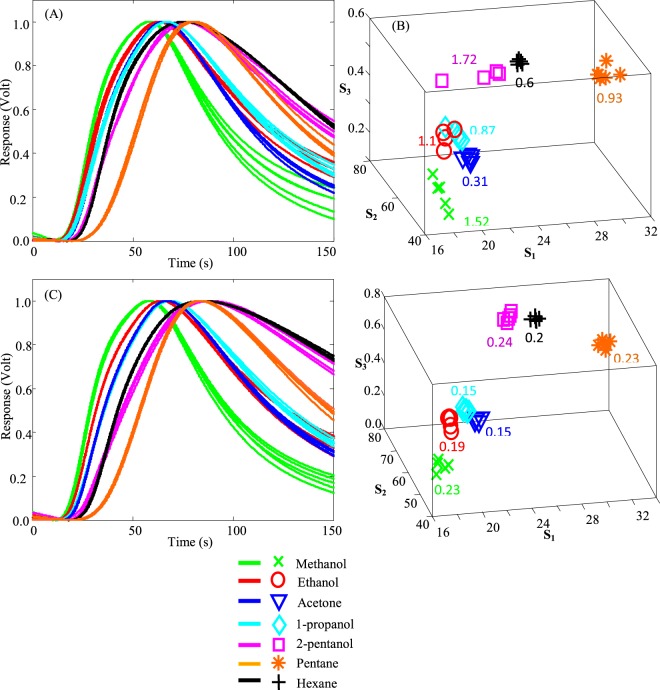
The normalized transient responses of the (**A**) Detector O, and (**C**) Detector X to 1000 ppm of methanol (green), ethanol (red), 1-propanol (cyan), 2-pentanol (magenta), acetone (blue), pentane (orange), and hexane (black). Each experiment is repeated 8 times. The feature space presentation for the seven examined analytes tested with the (**B**) Detector O, and (**D**) Detector. The values in (**B**,**D**) show the deviations between the 8 repetitions for each analyte.

The temporal responses obtained from the sensors are normalized between 0 to 1 (for ease of comparison) as discussed in^28^. The results are shown in Fig. 1A,C for the Detector O and X, respectively. Each experiment is repeated 8 times. For each detector, the response curves show that diffusion-physisorption procedure and accordingly the slopes of the curves during exposure and recovery change as the target gas changes. As it can be seen from Fig. 1A,C, these slopes are steeper for polar gases (e.g. methanol) as compared to non-polar gases (e.g. hexane). Also, the Detector X’s normalized responses are more distinct compared to those of the Detector O. In Fig. 1A,C, there might not be an obvious trend from polar to non-polar gases based on their relative polarity, however, by looking at the entire response time, the trend can be observed. For instance, if we look at the response level for final readouts of the sensor for different analytes, we can see there is a trend from methanol to hexane, as their final readout values increase from polar to non-polar analytes. This is also in agreement with the fact that diffusion is a passive and slow process, and hence the effects related to diffusion always occur at longer periods of time (as we see in the recovery part of the response curves).

To better visualize the selectivity capability of the detectors, a feature extraction method (described in^31^) is used to demonstrate the results in a 3D feature space. Three different features are extracted from each normalized transient response: These features include: 1) *S*_1_: the time at which the normalized response level reaches 0.05; 2) *S*_2_: the time at which the normalized response level reaches 0.95; and 3) *S*_3_: the magnitude of the normalized response at the final read out. The extracted feature vectors obtained from each set of transient responses are shown in Fig. 1B,D for the Detector O and X, respectively. The results shown in Fig. 1 demonstrate segregated clusters of feature vectors, representing the separation capability of the two detectors among different analytes. It is observed from the feature spaces (Fig. 1B,D) that the Detector X (coated with Cytonix) has a better separation capability as the clusters are concentrated with less overlap. For instance, ethanol and 1-propanol results in Fig. 1B show some overlaps, however, same gases (ethanol and 1-propanol) are segregated better in Fig. 1D which is for the Detector X. To compare quantitatively the selectivity of the two detectors (O and X) among different analytes, the 3D Euclidean distances of the average feature vectors (the mean of each feature components for each analyte) are calculated for each pair of the examined analytes in the feature space using Eq. (1):

1 $$D=\sqrt{{(Avg{S}_{1i}-Avg{S}_{1j})}^{2}+{(Avg{S}_{2i}-Avg{S}_{2j})}^{2}+{(Avg{S}_{3i}-Avg{S}_{3j})}^{2}}$$

In the above equation, *i*, *j* = *a*, *b*, *c*, *d*, *e*, *f*, or *g* refer to methanol, ethanol, 1-propanol, 2-pentanol, acetone, pentane, and hexane, respectively. The distances resulted from the interaction of each pair of analytes (from seven examined analytes) are listed in Tables 1 and 2 for the Detector O and X, respectively. As it is can be seen in Fig. 1B, ethanol cluster shows some overlaps with acetone cluster in the case of the Detector O. This is also confirmed from the related number to ethanol-acetone pair in Table 1, where the mean distance is small (2.91) which shows less selectivity compared to the same element for the ethanol-acetone pair in Table 2 (for the Detector X) which is 4.23. This value is ~45% more than that obtained for the Detector O. The largest mean distance in both tables is for the methanol-hexane pair which is attributed to the difference in their relative polarity numbers (see below). In essence, methanol is the most polar and hexane is the most non-polar analyte tested among all the tested analytes. Moreover, the average of the numbers listed in Table 2 for the Detector X is 12.45 which is ~43% more than the average of the mean distance listed in Table 1 for the Detector O (8.70).

**Table 1:** The Euclidean distances between the average feature vectors in the feature space for the Detector O (coated with Cr, Au, and Parylene C). The average of all Euclidean distances in this table is 8.70.

	a: ✕	b: ◯	c: ◊	d: □	e: ▽	f: ✚	g: ✴
a: ✕	0.00	4.65	9.12	15.94	7.81	14.36	24.91
b: ◯	4.65	0.00	4.45	11.29	2.91	10.00	20.62
c: ◊	9.12	4.45	0.00	6.83	1.34	5.89	16.42
d: □	15.94	11.29	6.83	0.00	8.13	3.34	10.37
e: ▽	7.81	2.91	1.34	8.13	0.00	6.81	17.42
f: ✚	14.36	10.00	5.89	3.34	6.81	0.00	10.62
g: ✴	24.91	20.62	16.42	10.37	17.42	10.62	0.00

**Table 2:** The Euclidean distances between the average feature vectors in the feature space for the Detector X (coated with Cr, Au, Parylene C, and Cytonix). The average of all Euclidean distances in this table is 12.45.

	a: ✕	b: ◯	c: ◊	d: □	e: ▽	f: ✚	g: ✴
a: ✕	0.00	8.78	11.00	25.33	10.12	22.09	28.92
b: ◯	8.78	0.00	10.82	23.25	4.23	21.42	28.64
c: ◊	11.00	10.82	0.00	14.33	1.38	11.35	18.45
d: □	25.33	23.25	14.33	0.00	15.26	4.53	7.12
e: ▽	10.12	4.23	1.38	15.26	0.00	12.00	18.96
f: ✚	22.09	21.42	11.35	4.53	12.00	0.00	7.22
g: ✴	28.92	28.64	18.45	7.12	18.96	7.22	0.00

### Effects of channel coating and analyte polarity

**Figure 2:**
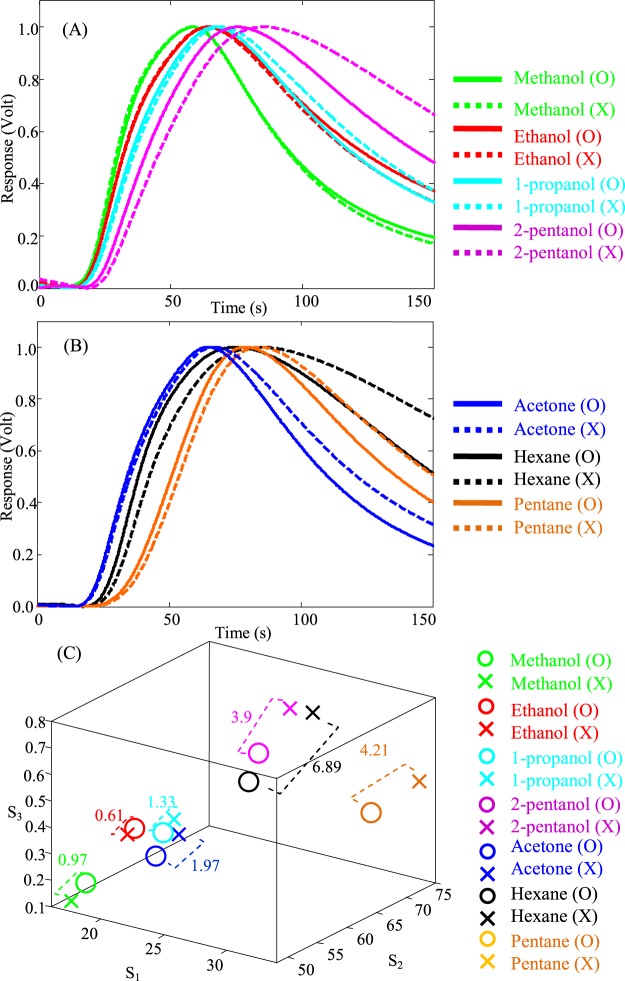
(**A**) The normalized transient responses of the Detector O (solid lines) and the Detector X (dash lines) to 1000 ppm of methanol (green), ethanol (red), 1-propanol (cyan), 2-pentanol (magenta). (**B**) The normalized transient responses of the Detector O (solid lines) and the Detector X (dash lines) to 1000 ppm of acetone (blue), pentane (orange), hexane (black). (**C**) The feature space presentation for the Detector O (shown with O markers) and Detector X (shown with X markers) for 7 tested analytes.

After comparing the detectors in terms of their selectivity between different analytes, it is also valuable to see how changing the polarity of the coating layer influences the temporal responses of the sensor to polar and non-polar analytes. In other words, the normalized temporal responses of two sensors to the same target gas are compared to see the effect of the channel and analyte polarities and their interaction (dipole-dipole interaction between the analyte and channel surface). The normalized transient responses of the Detector O and X to polar and non-polar analytes are shown in Fig. 2A,B, respectively. The extracted features from each normalized response for the detectors are presented in Fig. 2C. As it can be seen, the order of the feature vectors in the feature space changes by moving from the polar analytes to non-polar ones for the two the detectors. This can also be seen in the temporal responses (Fig. 2C). The Detector O (with higher polarity) shows less resistance to non-polar analytes compared to the Detector X (see Fig. 2A). For instance, it can be seen in Fig. 2A that diffuse-in and -out processes for methanol happen slightly faster in the Detector X with less polarity (shown with green dash line) compared to the slopes of diffuse-in and -out process for the Detector O with higher polarity (shown with the green solid lines). On the other hand, for non-polar analytes such as hexane (see Fig. 2C), this order changes in the temporal responses of the two detectors, where the Detector O with higher polarity (e.g. the black solid line for hexane) shows faster diffusion-in and -out and eventually faster retention time compared to the Detector X with less polarity of the channel surface material (e.g. the black dash line for hexane). This is due to “like dissolves like” principle^37^: the channel surface with higher polarity (the Detector O) shows a higher adsorption rate from the polar gases; whereas the channel with lower polarity (the Detector X) shows a higher adsorption rate from the non-polar analytes. As chemists say “like dissolves like”, meaning, for instance, polar solvents usually dissolve polar materials. Materials which are significantly different from a chemistry perspective (e.g. oil and water) do not mix and have a rather large interfacial energy difference. It is (in reality) more than just the polarity or chemical dipoles of the two materials (other factors include, but not limited to, hydrogen bonding and dispersive effects), but the polarity is a major part of this interaction^37^.” As a result, if the polarity of the channel coating material and compound are similar, the retention time increases (physisorption increases), as the compound interacts stronger with the channel surface. Therefore, polar compounds have long retention times on polar channels and shorter retention times on non-polar channels.

Changing the channel coating from the Detector O to X (more polar to less polar) has insignificant effects on polar analytes, especially on the ones with a smaller hydro-carbon chain and higher polarity. Among the four tested alcohols, 2-pentanol (least polar alcohol) shows the largest difference in the temporal responses of the two sensors (see Fig. 2A), which means changing the channel polarity affects the polar analytes less. On the other hand the two detectors response differently to less polar and non-polar gases such as acetone and alkanes (e.g. pentane and hexane, see Fig. 2B). This has also been projected in the feature space, where the feature vectors of the Detector O (presented with O markers) and the feature vectors of the Detector X (presented with X markers) and their 3D Euclidean distances are shown. As it can be seen, the distances between the feature vectors of the two detectors in response to the non-polar gases are larger (e.g. 6.89 for hexane) as compared to the polar ones (e.g. 0.61 for ethanol). Therefore, the results in Fig. 2 show larger differences between the two fabricated detectors in their responses to the non-polar gases as compared to the polar ones. This is attributed to the higher diffusion coefficient of polar gases which makes the diffusion part of diffusion-physisorption to be more effective. In other words, for the polar gases, diffusion is the dominant term in the diffusion-physisorption equation which makes the effect of channel coating (which has more influence on adsorption) less significant. On the other hand, the non-polar gases with lower diffusion coefficients have more time to interact with the channel surfaces, and hence, are more influenced with the channel surface material. Although diffusion rate of different gases is a significant parameter in device discrimination ability to distinguish different analytes, it is not the only parameter involved. As an example, ethanol and acetone have similar diffusion coefficients (~0.11 cm^2^/s). Therefore, if the diffusion rate was the only parameter for discriminating these two gases, the two detectors should have shown the same responses against these two gases and fail to distinguish between them. However, as it can be seen from Fig. 1, the Detectors O and X can distinguish between these two gases. Moreover, as it can be seen from Fig. 2, the two detectors show a more significant difference against acetone (1.97) rather than ethanol (0.61). This is also related to their polarity and the fact that changing the channel coating has more influence on less polar gases (such as acetone) rather than polar ones (such as ethanol). This is an obvious indication of the fact that the analyte discrimination in the microfluidic gas detectors is not a purely diffusion-based process, and there are analyte/channel surface-related parameters involved in enhancing/impeding sensor selectivity. As indicated in Fig. 2C, the difference between the feature vectors of 2-pentanol is 3.9, which is the largest among all the other alcohols and it is even higher than some of the less polar gases (such as acetone for which the difference between the feature vectors is 1.97). Comparing the diffusion coefficient of these two gases also justifies these numbers: acetone has a higher diffusion rate than 2-pentanol. In the next section, the surface free energy of the two fabricated channels (O and X) are estimated to quantify the interaction between the analyte and channel coating and its relation to the sensor discrimination power.

### Channel surface free energy

To determine the channel surface free energy of the two fabricated detectors, Owens, Wendt, Rabel and Kaelble (OWRK) method^38^ is used. The contact angle values of five of the tested analytes (methanol, ethanol, acetone, pentane, and hexane (as the representatives of the three families of alcohol, ketone and alkane)) on the channel surface of the two fabricated detectors are measured and listed in Table 3. The values represent the average of five measurements and the error presents the standard deviation.

**Table 3:** The contact angle measurement of five analytes on the surfaces of both detectors. The angles listed here are the averages of five measurements, and the error represents the standard deviation. The liquid-vapor (γ_LV_) and solid-vapor (γ_SV_) measured for both detectors are also listed here (the method of calculation of these values are explained at the end of this section).

Analyte	Contact angle on Detector O channel surface	Contact angle on Detector X channel surface	γ_SL_ for Detector O	γ_SL_ for Detector X
Methanol	13° ± 2	46° ± 3	0.28 ± 0.07	0.85 ± 0.1
Ethanol	16° ± 2	48° ± 2	2.07 ± 0.1	2.67 ± 0.09
Acetone	5° ± 1	46° ± 3	1.37 ± 0.06	0.09 ± 0.1
Pentane	10° ± 2	17° ± 1	5.11 ± 0.09	2.07 ± 0.06
Hexane	8° ± 1	16° ± 1	7.45 ± 0.06	0.08 ± 0.05

Based on the OWRK method, each of the interfacial tensions (liquid-vapor (*γ*_*LV*_) and solid-vapor (*γ*_*SV*_)) are broken down into two terms: polar surface tension (*γ*^*p*^) and dispersive surface tension (*γ*^*d*^) parts^39^ (see Eqs (2) and (3)).

2 $${\gamma }_{LV}={\gamma }_{LV}^{d}+{\gamma }_{LV}^{p}$$

3 $${\gamma }_{SV}={\gamma }_{SV}^{d}+{\gamma }_{SV}^{p}$$

The values for polar and dispersive liquid-vapor (*γ*_*LV*_) for the tested analytes are listed in Table 3. Combining Good’s and Young’s equations (Eq. (4)) and substituting Eq. (2) into it will result in Eq. (5):

4 $$\begin{array}{rcl}{{\rm{\gamma }}}_{{\rm{SL}}} & = & {{\rm{\gamma }}}_{{\rm{SV}}}+{{\rm{\gamma }}}_{{\rm{LV}}}-2\sqrt{{{\rm{\gamma }}}_{{\rm{SV}}}^{{\rm{d}}}{{\rm{\gamma }}}_{{\rm{LV}}}^{{\rm{d}}}}-2\sqrt{{{\rm{\gamma }}}_{{\rm{SV}}}^{{\rm{P}}}{{\rm{\gamma }}}_{{\rm{LV}}}^{{\rm{P}}}}\\ {{\rm{\gamma }}}_{{\rm{SL}}} & = & {{\rm{\gamma }}}_{{\rm{SV}}}+{{\rm{\gamma }}}_{{\rm{LV}}}\,\cos \,{\rm{\theta }}\end{array}\}$$

5 $$(1+\,\cos \,{\theta })(({{\gamma }}_{{LV}}^{{P}}+{{\gamma }}_{{LV}}^{{d}}/2\sqrt{{{\gamma }}_{{LV}}^{{d}}})=\sqrt{{{\gamma }}_{{SV}}^{{d}}}+\sqrt{{{\gamma }}_{{SV}}^{{P}}}\cdot \sqrt{{{\gamma }}_{{LV}}^{{P}}/{{\gamma }}_{{LV}}^{{d}}}$$

This equation can be simplified to a linear equation in the form of *y* = *A* + *Bx*, where

6 $$\begin{array}{rcl}{\rm{y}} & = & (1+\,\cos \,{\rm{\theta }})(({{\rm{\gamma }}}_{{\rm{LV}}}^{{\rm{P}}}+{{\rm{\gamma }}}_{{\rm{LV}}}^{{\rm{d}}})/2\sqrt{{{\rm{\gamma }}}_{{\rm{LV}}}^{{\rm{d}}}})\\ {\rm{x}} & = & \sqrt{{{\rm{\gamma }}}_{{\rm{LV}}}^{{\rm{P}}}/{{\rm{\gamma }}}_{{\rm{LV}}}^{{\rm{d}}}}\\ {\rm{A}} & = & \sqrt{{{\rm{\gamma }}}_{{\rm{SV}}}^{{\rm{d}}}}\\ {\rm{B}} & = & \sqrt{{{\rm{\gamma }}}_{{\rm{SV}}}^{{\rm{p}}}}\end{array}\}$$

**Figure 3:**
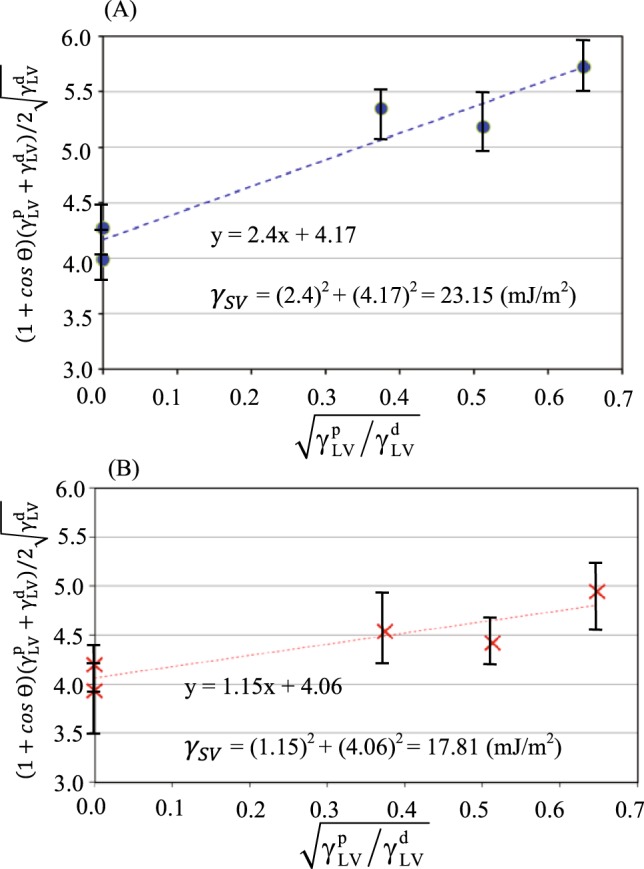
Owen-Wendt method is used for the determination of the surface free energy of two different channel coating surfaces for the (**A**) Detector O, and (**B**) Detector X.

After measuring the contact angles of different analytes on the both channel surfaces of the Detector O and X, the linear Eq. (5) is used to determine the solid surface tension of each of the fabricated channels. The results are shown in Fig. 3A,B for the Detector O and X, respectively. Each O or X marker in Fig. 3A,B presents the average value obtained from the five runs of contact angle measurements for each analyte. The error bars present the standard deviation from the average. The solid-vapor surface tension (*γ*_*SV*_) can then be measured from Fig. 3A,B for each particular surface. In essence, the line intercept (A) and slope (B) are the square roots of the dispersive and polar parts of the solid-vapor surface tensions, respectively. The results show that the solid-vapor surface tension (*γ*_*SV*_) for the channel surface of the Detector O (coated with Parylene C as the top layer) is 23.15 mJ/m^2^, and for the channel surface of the Detector X (coated with Cytonix as top layer) is 17.81 mJ/m^2^.

**Figure 4:**
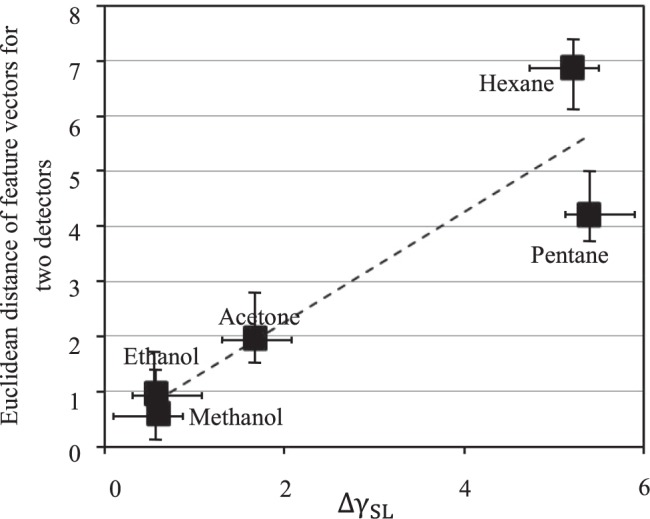
The linear relation between the Euclidean distances of the feature vectors of the two detectors vs. the difference between the surface tension of solid-liquid for the two detectors obtained for different analytes.

Using the Young’s equation (Eq. (4)), the solid-liquid surface tensions (γ_SL_) can then be estimated for each of the channel surfaces for different analytes. These results are listed in Table 3. Interestingly, the differences between the values of γ_SL_ for the two surfaces (the Detector O and X) are smaller for polar analytes (e.g. for methanol it is 0.56) and higher for non-polar analytes (e.g. for hexane it is 5.2). This was also observed in Fig. 2C, where the feature vectors of non-polar gases showed greater Euclidean distances for the two detectors, whereas the feature vectors for polar gases for the two detectors showed smaller Euclidean distances in the feature space. Figure 4 shows the linear relation between the distances of the feature vectors of the two detectors (shown in Fig. 2C) vs. the differences between γ_SL_ for the two channel surfaces of the two detectors (Δγ_SL_) for each of five tested analytes. This also shows as the surface of the channel changes the non-polar gases behave more differently than the polar ones. This is attributed to the fact that for the non-polar gases (with smaller diffusion rates) physisorption of the gas molecules to the channel walls is more dominant. As a result, the response of the Detector X against the non-polar gases is more than that of the Detector O.

## Conclusions

Two microfluidic-based gas detectors were fabricated with two different channel coating combinations (of layers) with different hydrophobicity. The selectivity of the two fabricated detectors among different analytes including: alcohols, ketones, and alkanes, were compared (both qualitatively and quantitatively) using a feature extraction method. The feature space presents that the Detector X (coated with Cytonix) has a better segregation power among the tested analytes compared to the Detector O. It has been shown that changing the polarity of the channel coating creates a more significant effect on the position of feature vectors of non-polar gases compared to polar ones. This is attributed to the higher diffusion rates of polar gases as compared to non-polar ones. This means that for the polar gases diffusion is the dominant term in the diffusion-physisorption equation which makes the effect of channel coating (which has more influence on adsorption) less significant. On the other hand, for the non-polar gases, lower diffusion coefficients result in having more time to interact with the channel surfaces, and hence, those are more influenced with the channel surface material. The comparison between the surface tensions of both channels showed that the difference in the solid-liquid surface for non-polar analytes is greater compared to polar ones. This supports the fact that changing the polarity of the channel coating alters more significantly the position of the feature vectors for non-polar analytes. These results show that when it comes to selecting the best channel surface coating material, the choice of non-polar coating surfaces offer more selectivity against non-polar gases, and in the case of polar gases this coating has less effects. This can be used to design an array of micro-channels with different polarities to increase the segregation power of the device.

## Methods

### Gas detector setup

**Figure 5:**
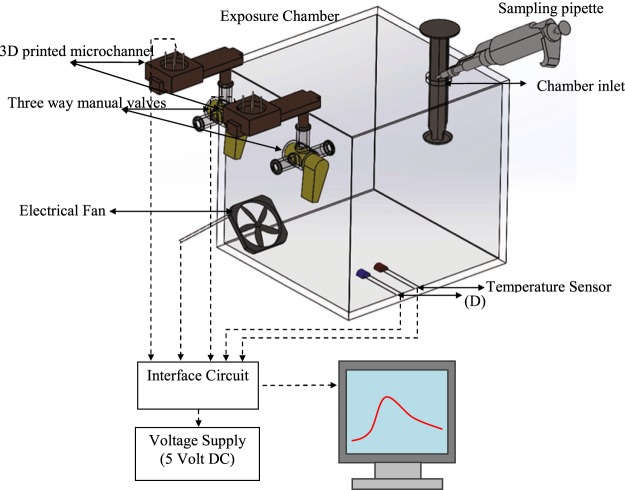
Schematic of the experimental setup. Two 3D-printed detectors are connected to the exposure chamber using three-way manual valves.

The experimental setup consists of three major parts (see Fig. 5): a one-liter Poly-methyl methacrylate (PMMA)-based gas chamber for sample injection (sampling chamber), two 3D-printed gas detectors with different coating materials (see below), and two 3-way manual valves connecting the detectors to the chamber or to clean air (laboratory environment). The detectors are made of VeroClear RGD810 material and connected to three way valves and placed on the top plate of the chamber. The PMMA chamber (which is fully sealed to prevent leakage of the gas molecules) is used as a gas sample injection container and for exposing the detectors to the target gases. To monitor the level of humidity and the temperature of the chamber, humidity and temperature sensors (Sensirion-SHT7x) are installed inside the chamber to record the ambient conditions. An electric fan is installed in one corner of the exposure chamber to create a uniform medium for the experiment and also faster recovery of the chamber between experiments. The chamber is dried in a vacuum oven (set at 80 °C) for two hours before using in the experiments.

### Fabrication process

#### Gas sensor

**Figure 6:**
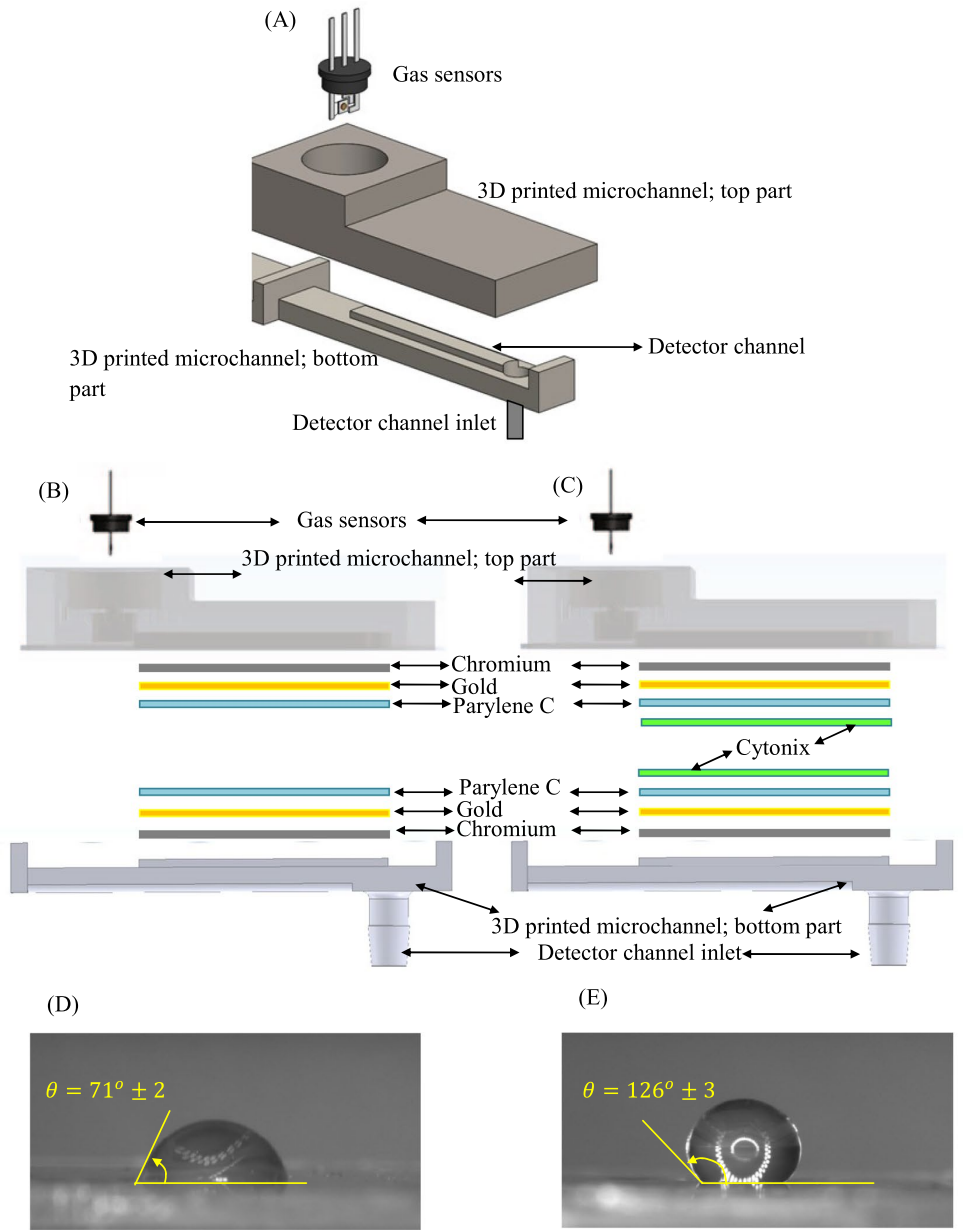
(**A**) Schematic of typical gas detector consisting a gas sensor and microchannel, (**B**) multilayer combinations of two fabricated detectors; (**B**) the Detector O: chromium, gold, Parylene C, (**C**) the Detector X: chromium, gold, Parylene C, and Cytonix. (**D**,**E**) Presents the contact angle values estimated for DI water on the surface of the Detector O and Detector X, respectively.

Each of the gas detectors consists of 3D-printed parts (which together create the microchannel) and a metal oxide semiconductor (MOS) gas sensor (FIGARO, TGS 2602) (see Fig. 6A, a typical schematic of the gas detector is shown). The detectors can be connected to sampling chamber or lab environment via the three-way valves.

#### Microchannel

The details of the fabrication process are reported in^28^. In essence, the microfluidic channel is coated with two different coating combinations (as it is shown in Fig. 6): (B) the Detector O includes three layers of chromium (35 nm), gold (65 nm), and parylene C (4 μm); and (C) the Detector X includes four layers of chromium (35 nm), gold (65 nm), and parylene C (4 μm), and Cytonix. The dimensions of the channel are kept the same in both detectors: *l* = 20 mm, *w* = 3 mm, *d* = 500 μm, where *l*, *w* and *d* represent the channel length, width and depth, respectively.

#### Channel hydrophobicity

To show the level of hydrophobicity of the channel surface, the contact angles of a droplet of deionized water (DI water) on both fabricated channel surfaces are estimated (see examples presented in Fig. 6D,E). Each contact angle is measured five times (using ImageJ), and the average and standard deviation were determined. Different surface treatments (resulting in different wettability) is attributed to the polarity of the top layer coated on the channel^40^.

#### Analytes

A set of experiments are performed using a number of VOCs with different polarities including: alkanes, ketones, and alcohols (which are mentioned from minimum to maximum polarity from left to right). A constant concentration (1000 ppm) of each of the analytes is injected into the system (for different experiments) using a precise micro-sampler (Pipet-Lite XLS). The concentration of the analyte is kept constant during all the experiments to eliminate the effect of the change in the analyte concentration on the detector response curves.

Table 4 lists the properties of the analytes tested here^41^. All the properties are related to each other. For example, as the hydro-carbon chain becomes larger in alcohols the molar mass increases, and on the other hand, diffusion coefficient and vapor pressure both decrease. Also, the larger the hydro-carbon chain the lower the polarity of the compound. This will result in having a smaller relative polarity number and larger boiling point. Similar trends are also seen among the ketone and alkanes.

**Table 4:** List of analytes tested here with their physical and chemical properties^41^.

Gas	Formula	Molar Mass [g/mol]	Diffusion Coefficient [cm^2^/s]	Vapor Pressure (20 °C) [mmHg]	Relative polarity	Boiling point [^o^C]	${{\boldsymbol{\gamma }}}_{{\boldsymbol{LV}}}^{{\boldsymbol{p}}}$ at 20 °C in mN/m	${{\boldsymbol{\gamma }}}_{{\boldsymbol{LV}}}^{{\boldsymbol{d}}}$ at 20 °C in mN/m
Methanol	CH_3_OH	32.04	0.1520	97.66	0.762	64.6	7	16.7
Ethanol	C_2_H_5_OH	46.07	0.1181	44.62	0.654	78.5	4.6	17.5
1-propanol	C_3_H_7_OH	60.1	0.0993	21.00	0.617	97.0	2.9	20.8
2-pentanol	C_5_H_11_OH	88.15	0.071	6.03	0.488	119.0	—	—
Acetone	C_3_H_6_O	58.08	0.1049	180.01	0.355	56.2	3.1	22.1
Pentane	C_5_H_12_	72.15	0.0856	429.78	0.009	36.1	0	16.2
Hexane	C_6_H_14_	86.18	0.0732	120.00	0.009	69.0	0	18.4

After six minutes, the sample is completely evaporated and uniformly spread into the chamber. The two detectors are then exposed (using the three-way valves) to the exposure chamber for 40 sec. The gas molecules start diffusing into the dead-end channels through the valves and reach the sensing pallets of the two sensors, which are placed at the other end of the channels. Finally, the detectors are connected to their original positions where they are exposed to the clean air again and the gas molecules diffuse out from the channels (i.e., referred to as the recovery stage). The kinetic responses of the gas diffusion along the channels are recorded (using an Arduino microcontroller) till *t* = 15 sec. This is long enough for the sensor to be recovered. The two detectors remain in this position for a few minutes before the sensors become fully recovered and ready for the next experiment. The experiments are all carried out at the room temperature of 25 ± 1 °C and relative humidity of 30 ± 5%. These conditions are kept constant during the experiments.
